# Role of Artificial Intelligence in Video Capsule Endoscopy

**DOI:** 10.3390/diagnostics11071192

**Published:** 2021-06-30

**Authors:** Ioannis Tziortziotis, Faidon-Marios Laskaratos, Sergio Coda

**Affiliations:** 1 Endoscopy Unit, Digestive Diseases Centre, Queen’s Hospital, Barking Havering and Redbridge University Hospitals NHS Trust, Rom Valley Way, Romford, London RM7 0AG, UK; johntziortziotis@gmail.com (I.T.); Sergio.coda@nhs.net (S.C.); 2 Photonics Group-Department of Physics, Imperial College London, Exhibition Rd, South Kensington, London SW7 2BX, UK

**Keywords:** capsule endoscopy, artificial intelligence, deep learning

## Abstract

Capsule endoscopy (CE) has been increasingly utilised in recent years as a minimally invasive tool to investigate the whole gastrointestinal (GI) tract and a range of capsules are currently available for evaluation of upper GI, small bowel, and lower GI pathology. Although CE is undoubtedly an invaluable test for the investigation of small bowel pathology, it presents considerable challenges and limitations, such as long and laborious reading times, risk of missing lesions, lack of bowel cleansing score and lack of locomotion. Artificial intelligence (AI) seems to be a promising tool that may help improve the performance metrics of CE, and consequently translate to better patient care. In the last decade, significant progress has been made to apply AI in the field of endoscopy, including CE. Although it is certain that AI will find soon its place in day-to-day endoscopy clinical practice, there are still some open questions and barriers limiting its widespread application. In this review, we provide some general information about AI, and outline recent advances in AI and CE, issues around implementation of AI in medical practice and potential future applications of AI-aided CE.

## 1. Introduction

Nowadays, artificial intelligence (AI) has been integrated into many daily activities, including social networking, banking, gaming, sports betting, weather, and retail. Face recognition on smartphones, detection of credit card frauds by banks, and personalised advertisements are only some examples of how AI is being used today. With specific regard to healthcare, AI offers a plethora of potential applications spanning detection, diagnosis, and monitoring of disease, prognosis prediction, and assessment of risk factors [1,2,3]. This is not a novel concept and there are relevant historical studies published even before the 1980s, for instance regarding the idea of computer-aided diagnosis [1]. In the last 20 years there has been an explosive interest in the field of AI in medicine and this is likely to change the way healthcare will be delivered in the future. Research and development of AI systems are continuous and have shown promising results in various medical specialties, such as the diagnosis of diabetic retinopathy [2] and skin cancer [3]. More specifically, in gastroenterology there has been significant progress over the last 20 years with a large and growing body of literature focusing on the role of AI particularly in the field of endoscopy.

## 2. Artificial Intelligence: General Information and Terminology

Artificial intelligence (AI) is the science of creating a machine or software that mimics human functions, such as learning and problem solving, in order to perform human tasks. The term was first used in 1956. There are many applications of AI in gastroenterology [4,5,6] (Table 1 [7]).

**Figure 1:**
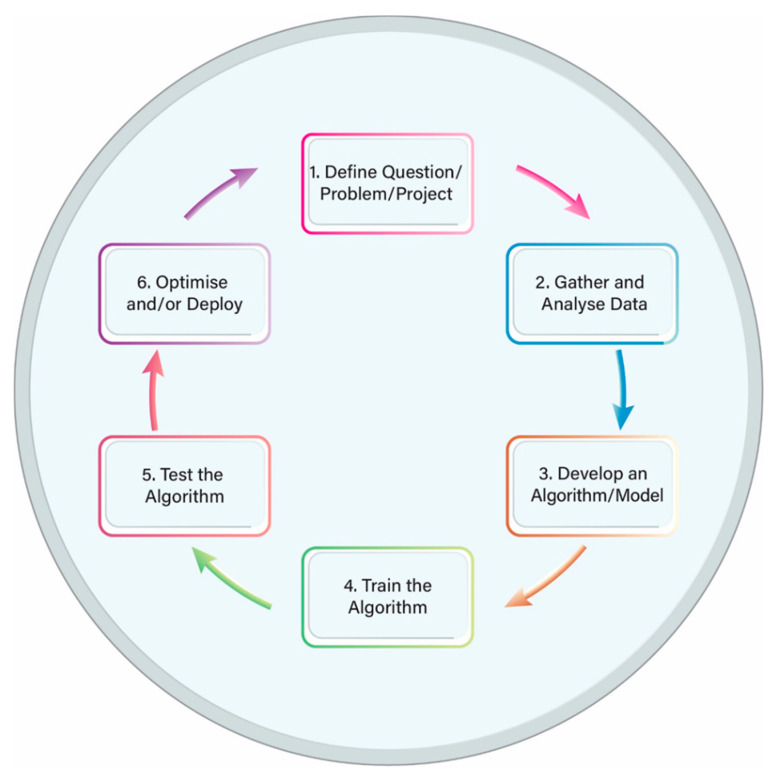
Machine learning life cycle.

Machine learning (ML) is a sub-field of AI that uses input data and mathematical algorithms to train a machine how to learn and improve without being explicitly programmed. The ML process includes several steps and works in a cyclic pattern: (a) first a question or a problem is defined; (b) then data are collected and the algorithm is trained; (c) next the performance of the algorithm is tested; and, finally, (d) a decision is made as to whether the performance is sufficiently good or further improvement is required. In the latter case, adjustments can be made and the cycle starts again (Figure 1). Table 2 shows some of the applications of ML in medicine [24].

**Figure 2:**
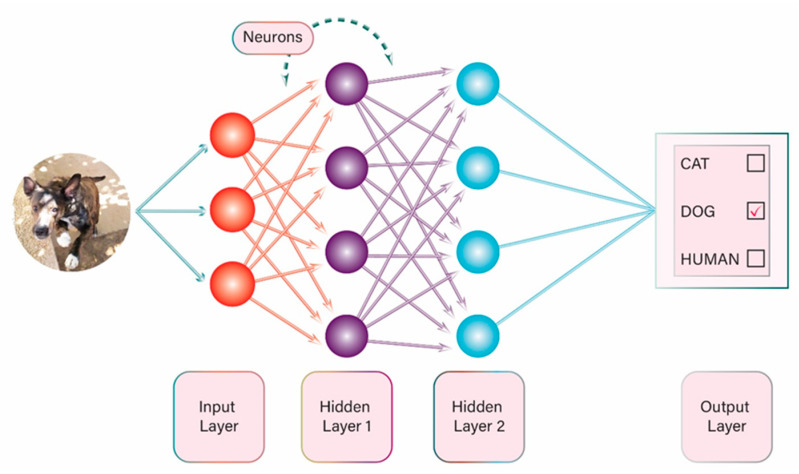
Example of artificial neural network.

There are many different ML methods and one of the most popular that has gained a lot of attention in recent years is the use of artificial neural networks (ANN). In this method of ML there are multiple interconnected layers of algorithms, which are programmed not only to process data in a specific pattern but also to feed data into each other in such a way that the system can be trained to carry out a specific task. The concept is based on the human brain function, with interaction among many neurons at multiple levels (e.g., synapses). ANNs can be used in image analysis: (a) they split an image into pixels; (b) the pixels are analyzed based on specific algorithms; (c) the data are combined and processed; and, finally, (d) the result is provided. The result might be either categorisation or classification of an object in the image (Figure 2) (e.g., identify a handwritten number or decide whether the person in the image is a man or a woman), or it might be detection of a specific feature, such as detection of a human in a picture or detection of an angiodysplasia in a small bowel capsule picture.

Another method used for ML is the support vector machine (SVM). SVMs are models for classifying sets of data by creating a line or plane to separate data into distinct classes. This allows the machine to then classify new input data based on previously input data [41,42].

**Figure 3:**
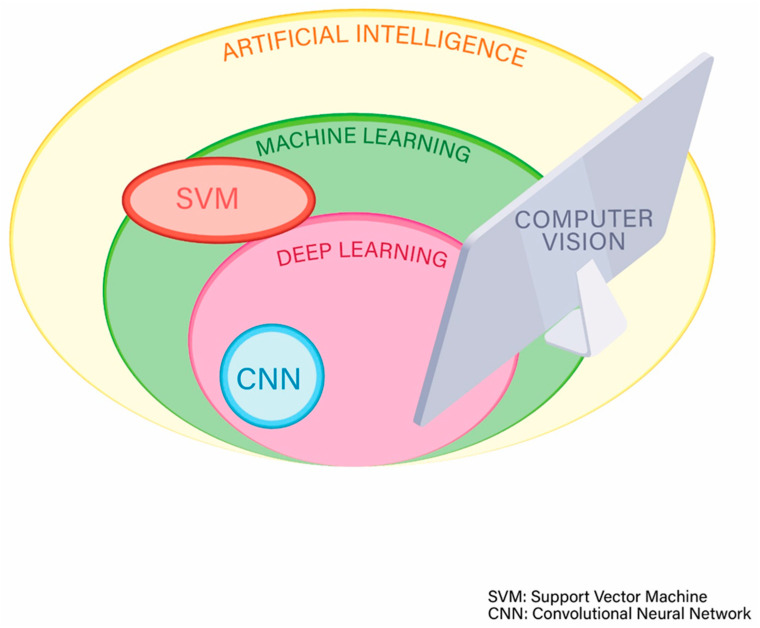
Subcategories of artificial intelligence.

Deep learning (DL) is a very popular technique of ML (Figure 3) that often uses multiple and complex ANNs and has the advantage that the more the volume of data are increased, the more the performance of the machine or computer is improved [43].

**Figure 4:**
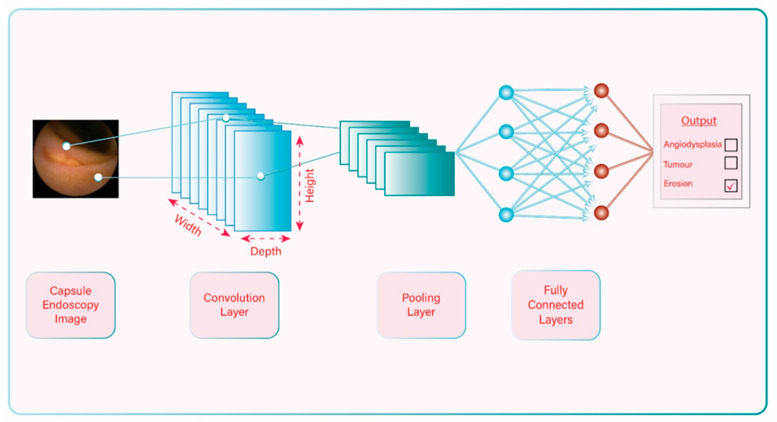
Example of convolutional neural network.

A convolutional neural network (CNN) is a DL algorithm that uses more advanced ANNs. Unlike the traditional ANN in which the layers of neurons are displayed in two dimensions, the CNN arranges its neurons in 3 dimensions (width, height, and depth) (Figure 4). In this way CNNs can extract features from an image and process the data in a more efficient way, and, as a result, their performance is typically better and faster. On the other hand, because of their complexity, one may need more time and more data to design, program, and train a CNN [44]. The amazing progress in CNNs in the last two decades has given an enormous boost to DL, and this sub-field of AI continues to expand.

As previously mentioned, the concept of AI is not completely new and has now existed for about 6 decades. However, problems with computing power, data storage, and graphic or image processing have hindered progress in AI until about 20–30 years ago. Technology advancement in the last few decades has helped overcome those obstacles and led to significant progress in the evolution of AI. Currently, data can be stored and analyzed in such a fast and efficient way that this has allowed training of computers to perform specific and complex tasks through data and image processing. Lastly, image analysis and processing using AI technology, is called computer vision and is a subtype of AI which also involves other sciences. Computer vision includes image recognition and image classification, and has numerous applications in human life. Almost every task requiring detailed image analysis may potentially benefit from AI and computer vision. It is obvious that the sub-specialty of gastroenterology that will benefit the most in the future is the one based on images, i.e., endoscopy, and of course this includes capsule endoscopy.

## 3. Progress of Artificial Intelligence-aided Diagnosis in Capsule Endoscopy and Pathology Detection

Capsule endoscopy (CE) is based on images for the diagnosis of gastrointestinal pathology and recently there has been remarkable progress in the field of CE with the application of computer vision [45] and AI. Many researchers across different countries worldwide have published interesting and highly promising results. The rationale of using AI to assist clinicians in their daily practice in CE is simple: to train a machine or program to analyse capsule endoscopy images, detect abnormalities, and define the diagnosis. It is crucial that clinicians are aware of the progress that has been made so far and understand the problems related to this new technological application, in order to realise the current status and full potential of AI in the future.

In the last 20 years, and mainly in the last 10 years, scientists and clinicians around the world have endeavoured to use AI technology to train algorithms on capsule endoscopy images or videos, in order to design systems for AI-aided CE diagnosis. These new AI computing systems are able to detect various common pathologies:

### 3.1. Ulcer/Erosions

Aoki et al. [46] trained a CNN system using 5360 CE images and assessed its performance using an independent test set of 10,440 small-bowel (SB) images including 440 images of erosions and ulcerations. The trained CNN required 233 s to evaluate 10,440 test images. The area under the curve (AUC) for the detection of erosions and ulcerations was 0.958. The sensitivity, specificity, and accuracy of the CNN were 88.2%, 90.9%, and 90.8%, respectively, at a cut-off value of 0.481 for the probability score.

Alaskar et al. [47] performed an interesting research study on the use of a CNN for the detection of ulcers in CE images. Interestingly, instead of developing a new CNN the authors used and compared two existing pre-trained CNN architectures: GoogleLeNet and AlexNet. A pre-trained network has pre-trained weights, which can be used in a related task. For the task of identifying ulcers the training was performed on 421 CE images and the test was performed on 105 CE images. Three learning rate values were explored, i.e., (0.01, 0.001, 0.0001), in order to evaluate the most appropriate setting. For GoogleLeNet the accuracy was 100%/97.143%/76.19% and the AUC was 1/0.9864/0.50 for learning rates 0.0001/0.001/0.01, respectively. For AlexNet the accuracy was 100%/100%/76.19%, and the AUC was 1/1/0.50 for learning rates 0.0001/0.001/0.01, respectively.

In 2016, Charisis and Hadjileontiadis [48] developed a novel algorithm to extract features from CE images in order to detect Crohn’s disease (CD) inflammatory lesions. Firstly, a hybrid adaptive filtering (HAF) process was proposed that aimed to refine the CE images, prior to feature extraction, by selecting via a genetic algorithm approach the most informative curvelet-based components of the images. Secondly, differential lacunarity (DLac) was employed for extracting color-texture features. The resulted scheme, namely HAF-DLac, incorporated support vector machines (SVM) for lesion detection. For the training of the algorithm, 400 frames depicting CD-related lesions and 400 lesion-free frames were acquired from 13 patients who undertook a CE examination. To validate the efficacy of the proposed scheme, two open CE databases were engaged, namely CapsuleEndoscopy.org (CaEn) [49] and KID [50,51]. The detection rates (accuracy, sensitivity, specificity, and precision) of severe, clearly defined lesions were 93.8%, 95.2%, 92.4%, and 92.6%, respectively.

Klang et al. [52] developed and evaluated a CNN for automated detection of SB ulcers in patients with Crohn’s disease. Their dataset included 17,640 CE images from 49 patients: 7391 images with mucosal ulcers and 10,249 images of normal mucosa. Results of the networks were compared for randomly split images and for individual patients. For randomly split images results were excellent, with AUCs of 0.99 and accuracy ranging from 95.4% to 96.7%. For individual patient-level experiments, the AUCs were also excellent (0.94–0.99).

CE can be used not only for diagnosis of CD but also for assessment of disease severity or for re-assessment in the case of flare-up. Although detection of inflammatory lesions in CE studies may be relatively easy, the assessment of disease severity or the comparison between a recent and an older CE study of the same patient can be very challenging. The Lewis score and the Capsule Endoscopy Crohn’s Disease Activity Index (CECDAI) are two small bowel CE indices that can be used for quantification of small bowel inflammation in CD. Although these indices have been validated, they are not simple to calculate which makes it difficult to use them routinely in everyday practice. Theoretically, AI could be a useful tool to assess disease severity as it could quantify the detected lesions in a fast, reliable, and reproducible way. Quite surprisingly, studies that used AI to assess CD severity in small bowel CE examinations are very limited. In 2012, Kumar et al. [53] described a methodology for CD lesion detection and CD lesion severity assessment in CE images, based on the use of SVMs. Their dataset included 533 CE images taken from 47 patient studies (30 patients contained CD lesions). The 533 CE images were annotated by an expert and three severity classes were studied: normal (*n* = 212), mild (*n* = 213), and severe (*n* = 108). The developed methodology showed high agreement with severity ratings manually assigned by an expert, and good precision (>90% for lesion detection) and recall (>90%) for lesions of varying severity. In a more recent study, Barash et al. [54] described a DL algorithm for automated grading of CD ulcers on CE. The authors developed a CNN and compared its performance in grading CD ulcers (grade 1 = mild, grade 2 = moderate, grade 3 = severe) with human performance. In experiment 1, two CE readers graded 1108 pathological CE images from 1 to 3. The overall agreement between the 2 experts was 31%. For differentiation between grade 1 and grade 3 ulcers the agreement was 76%, between grade 1 and grade 2 was 40%, and between grade 2 and grade 3 was 36%. In experiment 2, a consensus reading by three CE readers was performed on 1490 images that were used to train and test the CNN (1242 for training and 248 for testing). The overall agreement between the consensus reading and the automatic algorithm was 67%. The accuracy when comparing grade 1 to grade 3 ulcers was very good (91% agreement with AUC = 0.958, specificity = 0.91, and sensitivity = 0.91). When comparing grade 2 to grade 1 and grade 2 to grade 3 ulcers the performance was less accurate with overall agreement 65% and 79%, respectively. The CNN could accurately differentiate mild from severe CD ulcers whereas distinction between moderate and mild or severe CD lesions was less accurate.

### 3.2. Small Bowel Bleeding and Angioectasias

With regard to detection of small bowel angioectasias, Leenhardt et al. [55] developed a CNN algorithm and used 600 images for training and 600 images for testing. All images were selected from a large database called CAD-CAP [56]. The algorithm yielded a sensitivity of 100%, a specificity of 96%, a positive predictive value of 96%, and a negative predictive value of 100%. The reading process for an entire SB-CE video took on average 39 min.

In a different study, Tsuboi et al. [57] developed and validated a CNN to automatically detect angioectasias in CE images. They used 2237 CE images of angioectasias from 141 patients to train the CNN and an independent set of 10,488 SB images (including 488 images of SB angioectasias) from 20 patients to test the CNN. The AUC for the detection of angioectasias was 0.998. Sensitivity, specificity, positive predictive value, and negative predictive value of CNN were 98.8%, 98.4%, 75.4%, and 99.9%, respectively, at a cut-off value of 0.36 for the probability score.

In reference to detection of blood content in CE images, Aoki et al. [58] developed and tested a CNN which was compared to suspected blood indicator (SBI), a conventional tool used to automatically tag images depicting possible bleeding in the reading system. In order to train the CNN, the authors used 27,847 CE images (6503 images depicting blood content from 29 patients and 21,344 images of normal mucosa from 12 patients); while for validation they used an independent test set of 10,208 small-bowel images (208 images depicting blood content and 10,000 images of normal mucosa). The AUC for the detection of blood content was 0.9998. The sensitivity, specificity, and accuracy of the CNN were 96.63%, 99.96%, and 99.89%, respectively, at a cut-off value of 0.5 for the probability score, which were significantly higher than those of the SBI (76.92%, 99.82%, and 99.35%, respectively). The trained CNN required 250 s to evaluate 10,208 test images.

### 3.3. Protruding Lesions

In order to deal with the difficult task of detecting SB tumours in CE images, Barbosa et al. [59] proposed an algorithm that used information combined from both color and texture fields of CE images. The proposed textural features were then used as the input of a classifier based on ANNs. The experimental dataset contained 700 frames labelled as pathological (i.e., tumoural) and 2300 frames labelled as normal. The performance of the algorithm in classifying the images as pathologic or normal was excellent with 93.1% specificity and 93.9% sensitivity.

In a recent study, Saito et al. [60] developed and tested a computer-aided system based on a CNN. To train the CNN, 30,584 CE images of protruding lesions from 292 patients were used and an independent set of 17,507 test images from 93 patients, including 7507 images of protruding lesions from 73 patients, were used to test the CNN. The analysis of the 17,507 images was performed in 530,462 s. The AUC for the detection of protruding lesions was 0.911. The sensitivity and specificity of the CNN were 90.7% and 79.8%, respectively, at the optimal cut-off value of 0.317 for probability score. In individual patient analyses (*n* = 73), the detection rate of protruding lesions was 98.6%.

### 3.4. Early Detection of Prolonged Gastric Transit Time

One of the problems with CE is the occasional risk of prolonged gastric transit time which may render the examination incomplete due to premature battery exhaustion. It is generally important to know whether the capsule has not entered the jejunum after 2 h from the time of ingestion as an intervention may be needed, such as administration of a prokinetic drug. Although this can often be determined by experienced capsule endoscopy nurses or physicians on review of the real-time viewer, it would be certainly helpful for staff who are less familiar with interpreting the CE images. Gan et al. [61] developed and validated a CNN-based method for automatic retention-monitoring of the CE in the stomach/duodenal bulb. They used 180,000 CE images for training and 20,000 independent CE images for testing. The AUC for distinguishing the descending segment of duodenum was 0.984. The sensitivity, specificity, positive predictive value, and negative predictive value of the CNN were 97.8%, 96.0%, 96.1%, and 97.8%, respectively, at a cut-off value of 0.42 for the probability score.

### 3.5. Celiac Disease

Several studies have been performed in recent years for classification of celiac disease using AI technology [62]. Zhou et al. [63] established a deep CNN for quantitative measurement of the existence and degree of small bowel pathology in patients with celiac disease. The authors used the known 22-layer GoogLeNet architecture for this task. CE clips from 6 celiac disease patients and 5 controls were pre-processed for training and then clips of CE from 5 additional celiac disease patients and 5 additional control patients were used for testing. The CE videos of the small bowel were obtained by the PillCamSB2 video capsule system. A quantitative measurement for severity level, which the authors termed “evaluation confidence” (EC), was introduced in this study. The trained GoogLeNet was able to distinguish the frames from CE clips of celiac disease patients compared to controls and the results according to this evaluation confidence were found to be highly promising, achieving 100% sensitivity and specificity in the testing set. The t-test confirmed the evaluation confidence was significant enough to distinguish celiac disease patients from controls. Limitations of this study included the small number of patients and the older version of PillCamSB2.

### 3.6. Intestinal Hookworm Infection

In the Northern Hemisphere, intestinal hookworm infection may be rare but in many developing countries it is a common infection and constitutes a significant burden to healthcare systems with considerable morbidity and mortality for both children and adults. He et al. [64] proposed a novel methodology for hookworm detection which combines two CNNs: one to extract the edge features and the second to classify the hookworms. The proposed framework was evaluated using a dataset of 440,000 CE images of 11 patients (age 14–74) with intestinal hookworm infection that were collected from the West China Hospital. This methodology achieved good results with sensitivity 84.6%, specificity 88.6%, accuracy 88.5%, and ROC-AUC 0.895.

### 3.7. Multiple Lesion Detection

Iakovidis et al. [65] assessed the validity of an automatic lesion detection software in CE which was based on color pattern recognition. The authors used 137 de-identified CE single images, 77 showing pathology, and 60 normal images. The average performance, in terms of the area under the receiver-operating characteristic curve (AUROC), reached 89.2 ± 0.9%.

Based on the concept that when endoscopy images are closely analysed each abnormality shows a unique texture that can be distinguishable from normal ones, Nawarathna et al. [66] (2014) proposed a multi-texture analysis method for multiple abnormality detection in endoscopy videos. Their method was designed to detect erythema, blood, polyp or ulcer/erosion and was tested on both colonoscopy and CE images. For the evaluation of CE abnormal image detection, the authors used 100 abnormal images (25 from each texture) and 400 normal images, and reported a sensitivity of 92% and specificity of 91.8%. Although the results of these two studies were satisfactory, there were several limitations, including an insufficient number of CE images to reliably evaluate the diagnostic accuracy (a usual CE study contains approximately 50,000–60,000 frames).

In a different study, Iakovidis et al. [67] presented a novel three-phase CNN-based methodology for automatic detection and localisation of lesions in CE images. In the first phase, video frames were classified into normal or abnormal; in the second phase the CNN suggested the possible locations of lesions detected in CE images; and in the third phase a new algorithm localised the lesions in CE images. This methodology was tested on various datasets obtained from the known KID dataset [52] and when it was tested on a CE video (named “Case 1”) of the KID database, it achieved an AUC of 0.886 for anomaly detection, and an AUC of 0.769 for anomaly localisation.

More recently, Ding and Shi et al. [68] validated a CNN-based algorithm for the identification of abnormalities in SB-CE images. The authors collected 113,426,569 images from 6970 patients who had SB-CE at 77 medical centers from July 2016 through July 2018. A CNN-based auxiliary reading model was trained to differentiate abnormal from normal images using 158,235 SB-CE images from 1970 patients. Images were categorised as normal, inflammation, ulcer, polyps, lymphangiectasia, bleeding, vascular disease, protruding lesion, lymphatic follicular hyperplasia, diverticulum, parasite, and other. The model was further validated in 5000 patients (no patient overlap existed with the 1970 patients in the training set). The same patients were evaluated by conventional analysis and CNN-based auxiliary analysis by 20 gastroenterologists. If there was agreement in image categorisation between the conventional analysis and CNN model, no further evaluation was performed. If there was disagreement between the conventional analysis and CNN model, the gastroenterologists re-evaluated the image to confirm or reject the CNN categorisation. The CNN-based auxiliary model identified abnormalities with 99.88% sensitivity in the per-patient analysis and 99.90% sensitivity in the per-lesion analysis. Conventional reading by the gastroenterologists identified abnormalities with 74.57% sensitivity in the per-patient analysis and 76.89% in the per-lesion analysis. The mean reading time per patient was 96.6 ± 22.53 min by conventional reading and 5.9 ± 2.23 min by CNN-based auxiliary reading (*p* < 0.001).

Publications in the last few years have shown that there are AI systems that can detect multiple lesions in CE images with very good performance. Clearly the next step should be to design an AI system that could not only detect the lesions but also categorise them, practically meaning that it could provide a diagnosis. To our knowledge no such system exists to date that has been tested thoroughly and has shown good and reliable results.

There is no doubt that there has been remarkable progress in the use of AI algorithms for CE diagnosis. However, many of the studies or databases published in the last decade have some significant limitations:

- Some studies have low numbers of frames/videos (mainly the older studies) and this is limiting the statistical power of the results. Nevertheless, in recent years some centers have created large databases allowing more reliable results in their studies. It seems that we now have enough data available for processing and this is important for future studies.
- Frames with inadequate bowel preparation or reduced image quality were not included in some studies [53] or it is not known if they were included in others. This may affect false-positive or false negative results.
- Only pictures from a single CE system or version were used in most studies [47,59,64,66]. This factor may limit a more widespread application of the algorithm. An algorithm that was designed using images and videos from one CE system may not have the same reliability if used in a database with images from a different CE system or version.
- The large majority of the studies published up to date were designed to assess for specific pathology, e.g., the algorithm detects only blood or ulcers in the small bowel. This is a significant limitation because clinicians are looking for a specific pathology only in a few cases, for example to assess disease activity in a patient with diagnosed Crohn’s disease. More commonly, the etiology of symptoms is unknown, and so it would be extremely useful to have an algorithm that could detect various types of lesions in the small bowel. Some researchers have worked towards this direction.

## 4. Implementation of Artificial Intelligence in Capsule Endoscopy

The advantages of AI in CE diagnosis include:

- Time efficiency: conventional human reading of a complete small bowel CE video can take from 30 to 90 min, approximately. AI algorithms are much faster as they can “read” a complete CE video in <30 min and in some cases in <10 min [68,69];
- Error reduction due to human limitations such as biases, fatigue or inexperience;
- Improvement of training and learning opportunities: AI technology can be used to provide clinicians with only abnormal CE images for review.

The disadvantages include:

- Cost;
- Need for large databases (CE images or videos). These databases are necessary for training and testing in order to increase sensitivity and specificity and achieve excellent results. It appears that in the last few years, large databases are being increasingly created in many centers around the world and this will not be a problem in the future.

Only a few hospitals around the world are using AI in everyday clinical practice to help doctors with lesion detection (this is called computer aided detection, CADe) or lesion categorisation and disease diagnosis (this is called computer aided diagnosis, CADx). In some centers, CADe/CADx is used as an experimental tool. In gastroenterology, perhaps the most well-known and useful CADe/CADx application is the development of systems that can detect (and in some cases further categorise) polyps either on selected videos or during live colonoscopy [9,10,70,71,72], and this has been implemented in clinical practice only recently. On the other hand, in the field of CE, the use of CADx in hospitals is at its embryonic stages. Since AI systems and algorithms are available to detect abnormal CE images, one may wonder why this technology is not yet implemented in CE clinical practice.

In general, it is difficult to predict when AI will come into widespread clinical application in CE and what its exact role will be. Some challenges are related to the AI system and include the following research questions and considerations:

- Is testing of the AI system with images and videos from an existing database adequate or should it be tested on real patient CE images/videos before it comes into practice? Some of the AI algorithms have been checked on real patient data but others have not;
- How can one be sure that a new AI algorithm will have minimal or no problems with overfitting and spectrum bias? These are two of the most common problems that AI engineers have to deal with when designing a new AI algorithm. Overfitting occurs when an algorithm becomes so accurate on a limited dataset that its predictions are not well generalised to new datasets. Most often the algorithm is overfitted to the training dataset. Spectrum bias occurs when the dataset used for the development of the algorithm does not adequately represent the range of disease manifestations or patients that will be encountered in clinical practice (target population) [73];
- What is the gold standard method that a new AI system should be compared to? Is it the conventional reading of CE by expert clinicians? Is it the conventional reading of CE by the clinicians of the hospital where the AI system will be implemented? Possibly, a different AI method will be the gold standard in the future;
- To date, all known studies are retrospective. Large prospective studies are warranted to validate new AI systems in CE. A multicenter, multinational, blinded prospective trial is currently evaluating the role of AI in small bowel capsule endoscopy for obscure GI bleeding (ClinicalTrials.gov Identifier: NCT04821349);
- Cost may be a limitation for widespread use.

On the other hand, there are some issues that are not related to the AI system but more to the intrinsic nature of medical practice:

- How will the AI system be used in day-to-day practice? For example, a company may have developed an AI system that can detect abnormal CE images with sensitivity of 95% when tested on real patient CE videos. Is the false-negative 5% rate acceptable and should clinicians review only the abnormal images detected by the system (AI-based auxiliary reading)? Conversely, is this sensitivity not acceptable and should clinicians review the whole CE video of patients in order to achieve a higher sensitivity (combination of AI +human reading)?
- How difficult is it to use these new AI systems? How long is the learning curve?
- How can we define malpractice after implementation of CADe/CADx?

## 5. The Future of Artificial Intelligence-Aided Capsule Endoscopy

The new AI systems that have been developed in recent years to help clinicians and assist CE interpretation are constantly improving. The ideal AI system for detection of pathology in CE videos should have the following characteristics:

- Excellent performance (high sensitivity/specificity/accuracy and very low error rate) in abnormal CE image detection;
- Ability to detect multiple lesions;
- Ability to categorise the lesions;
- Fast reading time of CE videos;
- Easy to use.

In this review article we focused on the progress of AI-augmented lesion recognition in CE images and videos. The future in CE is extremely interesting and promising and many advances have been made in other fields outside of AI [74,75,76], such as:

- Locomotion [77]. The ability to navigate the capsule within the bowel with the use of an internal or external system is very appealing and could help solve some difficult problems including prolonged capsule transit time in the stomach, insufficient lesion assessment and bowel stenosis. Several companies have expressed interest and have conducted experiments to design and study such a system. At present, the most promising technology uses a magnetic capsule with an external navigation system but is not yet widely implemented.
- Assessment of quality of bowel visualisation in capsule endoscopy [78,79,80];
- Capsule with biopsy properties [81];
- Capsule with therapeutic properties. This is also an area of interest for different companies and relevant examples of therapeutic interventions include guided drug delivery [82] and treatment of gastrointestinal bleeding [83];
- Capsule with improved technological characteristics. The new capsules that will be launched onto the market in the future will offer better quality of images (high resolution) and longer battery life.

Technology advancements will lead to new upgraded capsules with ability of navigation while AI will provide computing systems that will be able to screen CE videos, find the possible lesions and form a diagnosis in less than 30min. When this combination is introduced into everyday practice the impact on endoscopy will be tremendous. It will be possible to accurately and promptly investigate the whole gastro-intestinal tract with just one capsule. Whether this future is far or close is yet to be determined as the advent of this new technology depends on the interplay of several factors, including scientific, economical, and managerial.

## 6. Conclusions

In the near future, AI will play an increasingly important role in gastroenterological practice and especially in CE. AI is expected to offer multiple useful applications in GI disease risk stratification, lesion recognition and assessment, diagnosis and treatment. The progress in the last decade suggests that AI-aided CE will be available in the near future and will radically transform medical practice and patient care. Collaboration of AI engineers with clinicians is essential to achieve this goal. Given the emerging role of AI technologies and their application in CE, clinicians should be familiar with the basic concepts, the advantages and the limitations of AI, and the encouraging results of the currently available literature. Indeed, it is envisaged that many of the challenges described in this review will be overcome in the future with the advent of more sophisticated AI systems, larger databases, high-powered studies, and testing in real world data.

## Figures and Tables

**Table 1:** Artificial intelligence (AI) systems and related functions in gastroenterology.

AI System Categories	Areas of Assistance
Technical	Scope guidance for colonoscope insertion [8]
Detection (CADe)	Polyps detection [9,10] Bleeding detection * [11,12]
Diagnostic (CADx)	Early cancer identification [13,14] Cancer staging (estimation of invasion depth) [15,16] Polyp characterization or classification [17,18] Diagnosis of normal vs. inflammatory mucosa in IBD [19] GI disease prediction from patient data [20]
Therapeutic	Lesion delineation [13,21] Assistance in therapeutic decisions (such as complementary surgical resection post-endoscopic resection for malignant lesions) [22] Risk stratification, prediction of outcomes, and potential need for therapeutic intervention (in GI bleeding) [23]

* Mainly in small bowel exploration for obscure GI bleeding. AI: Artificial intelligence; CADe: Computer-assisted detection; CADx: Computer-assisted diagnosis; IBD: Inflammatory bowel disease; GI: Gastrointestinal.

**Table 2:** Machine learning applications in medical domains.

Medicine Domain	ML Applications	References
Radiology	Radiological imaging tasks such as: (i) Risk stratification. (ii) Therapy response. (iii) Lesions segmentation and classification. (iv) Multi-omics disease discovery. (v) Discovery of radiographic imaging biomarkers. (vi) Creating study protocols.	[25,26,27,28,29]
Pathology	Digital pathological image analysis notably: (i) Tissue phenomics. (ii) Histopathological imaging analysis. (iii) Whole Slide imaging analysis.	[30,31,32]
Oncology	Early cancer diagnosis and prognosis: (i) Cancer metastases detection. (ii) Molecular subtyping of cancer. (iii) Cancer detection from microarray gene expression data (iv) Risk classification of cancer survival.	[33,34,35]
Cardiology	Early detection of cardiovascular diseases based on: (i) Electrocardiographic interpretation. (ii) Echocardiography interpretation. (iii) Myocardial perfusion analysis. (iv) Discrimination of different diseases with similar symptoms like constrictive pericarditis and restrictive cardiomyopathy or hypertrophic cardiomyopathy and physiological hypertrophy.	[36,37,38]
Neurology	Neurological disorders identification and prediction: (i) Electroencephalography data interpretation (ii) Electromyography data interpretation (iii) Augmented Intelligence such as: (iv) Restoring the control of movement in patients with quadriplegia. (v) Controlling upper-limb prostheses via Brain-computer interface.	[39,40]
